# Expression analysis of lung miRNAs responding to ovine VM virus infection by RNA-seq

**DOI:** 10.1186/s12864-018-5416-0

**Published:** 2019-01-18

**Authors:** Martin Bilbao-Arribas, Naiara Abendaño, Endika Varela-Martínez, Ramsés Reina, Damián de Andrés, Begoña M. Jugo

**Affiliations:** 1 Department of Genetics, Physical Anthropology and Animal Physiology, Faculty of Science and Technology, University of the Basque Country UPV/EHU, 48080 Bilbao, Spain; 2 Institute of Agrobiotechnology (CSIC-UPNA-Government of Navarra), Avenida de Pamplona 123, 31192 Mutilva, Navarra, Spain

**Keywords:** Visna-Maedi, miRNAs, RNA-seq, Host-virus interaction, Differential expression

## Abstract

**Background:**

MicroRNAs (miRNAs) are short endogenous, single-stranded, noncoding small RNA molecules of approximately 22 nucleotides in length. They regulate gene expression posttranscriptionally by silencing mRNA expression, thus orchestrating many physiological processes. The Small Ruminant Lentiviruses (SRLV) group includes the Visna Maedi Virus (VMV) and Caprine Arthritis Encephalitis (CAEV) viruses, which cause a disease in sheep and goats characterized by pneumonia, mastitis, arthritis and encephalitis. Their main target cells are from the monocyte/macrophage lineage. To date, there are no studies on the role of miRNAs in this viral disease.

**Results:**

Using RNA-seq technology and bioinformatics analysis, the expression levels of miRNAs during different clinical stages of infection were studied. A total of 212 miRNAs were identified, of which 46 were conserved sequences in other species but found for the first time in sheep, and 12 were completely novel. Differential expression analysis comparing the uninfected and seropositive groups showed changes in several miRNAs; however, no significant differences were detected between seropositive asymptomatic and diseased sheep. The robust increase in the expression level of oar-miR-21 is consistent with its increased expression in other viral diseases. Furthermore, the target prediction of the dysregulated miRNAs revealed that they control genes involved in proliferation-related signalling pathways, such as the PI3K-Akt, AMPK and ErbB pathways.

**Conclusions:**

To the best of our knowledge, this is the first study reporting miRNA profiling in sheep in response to SRLV infection. The known functions of oar-miR-21 as a regulator of inflammation and proliferation appear to be a possible cause of the lesions caused in the sheep’s lungs. This miRNA could be an indicator for the severity of the lung lesions, or a putative target for therapeutic intervention.

**Electronic supplementary material:**

The online version of this article (10.1186/s12864-018-5416-0) contains supplementary material, which is available to authorized users.

## Background

The Small Ruminant Lentiviruses (SRLVs) are in a group of RNA viruses in the lentivirus genus that infect cells of the monocyte/macrophage lineage from sheep and goats. This infection causes progressive inflammatory lesions in the lungs, brain, mammary glands and joints that are characterized by lymphoid hyperplasia, interstitial infiltration of mononuclear cells and interstitial pneumonia. Visna/Maedi disease (VM) has a great economic importance derived from decreased animal production and increased replacement rates [1]. Infection is present in most countries that raise sheep but the impact on production and animal welfare is affected by breed [2] and flock management [3].

Not every infected animal shows the disease due to the importance of the host genetic background [4]. In genetic association studies several molecules have been shown to be related to VMV infection: Toll like receptors (TLRs), antiviral proteins (APOBEC family, TRIM5alpha, tetherin), and cytokines (among others) [5, 6]. To our knowledge, microRNAs (miRNAs) have not been analyzed in relation to this viral disease.

miRNAs are a class of noncoding endogenous RNAs of approximately 22 nucleotides that regulate gene expression posttranscriptionally. By binding to mRNA molecules and with the help of the RNA-induced silencing complex (RISC), they can silence or cleave mRNA molecules [7]. They are one of the most abundant gene expression regulators and have an effect on phenotypic variations in domestic animals [8]. Several studies have identified miRNAs in various sheep breeds, although miRBase 21 includes only 106 miRNA precursors and 153 mature sequences (January 2018). Regarding tissue types that have been previously studied, most of the work has focused on muscle quantity, wool quality, fertility and fat deposition [9–12] with little attention to animal health and welfare.

Viruses exploit host gene pathways to accomplish their basic biological processes, from transcription to protein synthesis, thus, ensuring their own survival. MicroRNA levels can be altered due to the host’s own immune response modulation [13]; however, viruses can also modulate the expression of host genes to avoid detection by the immune system or to modify cell survival pathways [14]. Furthermore, it has been proposed that host miRNAs can directly target RNA viruses either cleaving them or stabilizing them [15]. Another way that miRNA expression may change involves virally encoded miRNAs [16].

The aim of this study was to uncover the host mechanisms that are associated with VM disease in sheep. To this end, the cellular miRNAs differentially expressed at different stages of infection were identified, and information about involved genes, the mechanisms, and relevant pathways was inferred via bioinformatics analyses. These predictions could also contribute to uncover the roles of miRNAs in host-virus interactions.

## Methods

### Animals

Thirty Rasa Aragonesa adult (3 to 6 years) ewes were included in this study, in different stages of a natural infection of VMV. The samples were obtained from different commercial flocks in the routine of the Veterinary Faculty (University of Zaragoza) in the framework of the national research project ref. AGL2010–22341-C04–01. The complete experimental procedure was approved and licensed by the Ethical Committee of the University of Zaragoza (ref: PI09/10). Animals were euthanized by an intravenous injection of a barbiturate overdose (Dolethal®, Vetoquinol, Spain) and exsanguinated.

Animals were classified attending to their VMV infection status (seronegative or seropositive) using an Enzyme-Linked ImmunoSorbent Assay (ELISA) (ELITEST, Hyphen), and the clinical outcome (asymptomatic and diseased). For RNA-seq analysis, a total of 15 animals were included: Five animals were seronegative for VMV (seronegative group), five of the animals tested seropositive for VMV but did not show clinical symptoms (seropositive asymptomatic group) and, the remaining five animals were seropositive and had lung lesions (lesions group). For validation of the sequencing data 15 different animals were included (5 seronegative, 5 seropositive asymptomatic and 5 with pulmonary lesions) (Table 1).

**Table 1:** Samples used in RNA-seq and RT-qPCR study

RNA-seq	
Status	Animals (15)
Pulmonary lesions	1P, 2P, 7P, 9P, 10P
Seropositive asymptomatic	8P, 11P, 12P, P19, 4
Seronegative	7,10,11, 13,14
RT-qPCR	
Status	Animals (15)
Pulmonary lesions	P21, P22, P24, P25,P26
Seropositive asymptomatic	1, 2, 3, 5, 6
Seronegative	12, P-13, P-14, P-15, P-16

### Tissue collection, RNA extraction and small RNA sequencing

A sample from lung was aseptically taken from each animal and preserved in RNAlater solution (Ambion, Austin, TX, USA) at −80 °C until used. Total RNA was isolated from lung tissue using Trizol (Invitrogen, Carlsbad, CA, USA) extraction. 60–70 mg tissue samples were homogenized in 1 ml of Trizol using Precellys®24 homogenizer (Bertin Technologies, Montigny le Bretonneux, France) combined with 1.4 and 2.8 mm ceramic beads mix lysing tubes (Bertin Technologies). After adding chloroform, RNA was precipitated from the upper aqueous phase with isopropanol, washed with ethanol, suspended in RNase free water and stored at − 80 °C. RNA quantity and purity was assessed with NanoDrop 1000 Spectrophotometer (Thermo Scientific Inc., Bremen, Germany). RNA integrity and concentration was assessed with the 2100 Bioanalyzer (Agilent Technologies, Santa Clara, CA, USA).

The small RNA libraries were generated with Illumina’s TruSeq small RNA library preparation kit following manufacturer’s instructions. Sequencing was performed in CNAG-CRG core facility (Barcelona, Spain), using an Illumina HiSeq 2500 instrument. Single-end sequencing with 50 bp read length was used for miRNAs.

### Prediction of miRNAs

**Fig. 1:**
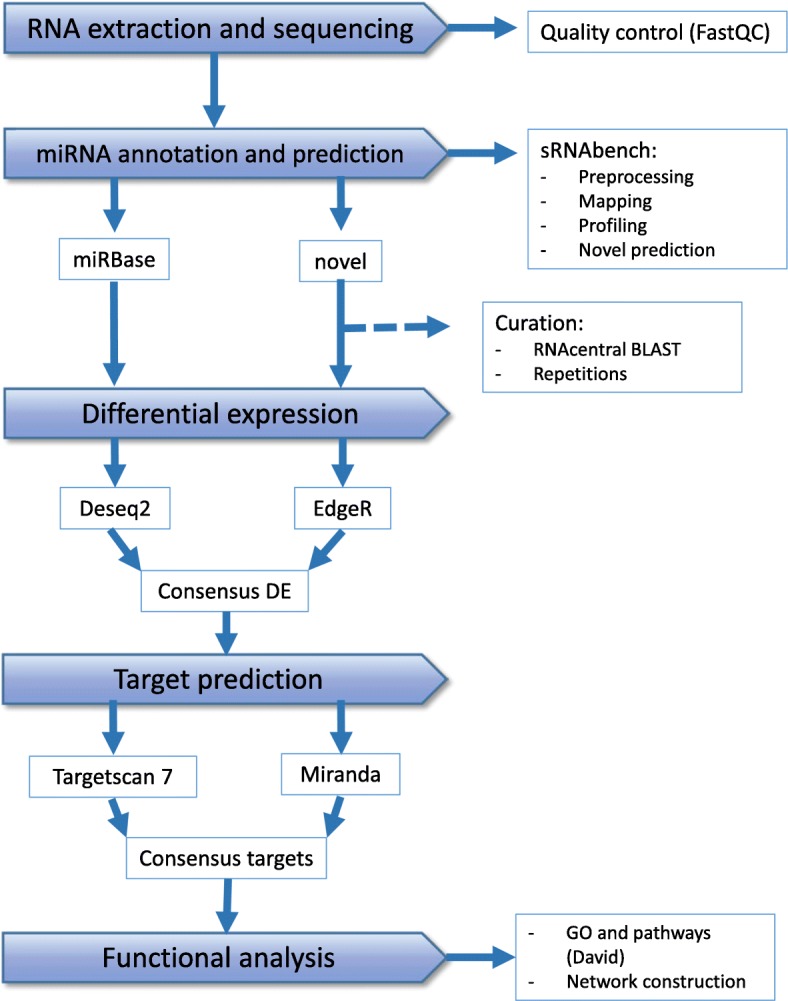
Computational pipeline of data analysis. The figure illustrates the four steps of the data analysis starting from the RNA extraction and sequencing: miRNA detection and prediction, differential expression, target prediction and functional analysis

The quality control was performed with fastQC and the following computational pipeline was followed (Fig. 1). Raw reads were analyzed with the sRNAbench web tool, which is included in the sRNAtoolbox collection of tools [17]. This program performed the preprocessing, mapping, expression profiling and novel miRNA prediction. Parameters were set to minimum read count of four, allowing one mismatch, with full read alignment and three species were selected to search for homologs: goat, cattle and mouse. After that, the prediction results of novel miRNAs were manually curated to remove repeated entries that just differed in one nucleotide and to give more updated miRNA names. Only miRNAs marked with high confidence by the program were selected for further analysis. Since the program only uses miRNAs present in miRBase, new predicted miRNAs that could had been previously described elsewhere were locally blasted against the whole RNAcentral database (http://rnacentral.org/) looking for perfect identity.

### Differential expression

Before the differential expression analysis, the matrix of novel miRNAs was built excluding repeated miRNAs that mapped in different places, miRNAs that appeared in less than half of the samples and with counts lower than ten. This was done following common criteria in the field to perform a conservative analysis. In addition, it was performed a principal component analysis (PCA) (Additional file 1) to check the grouping of the samples with the DESeq2 Bioconductor R package (https://bioconductor.org/packages/release/bioc/html/DESeq2.html). Three out of the 15 samples were excluded from further analysis - these outliers highly increased variability - leaving three groups with four samples each. DESeq2 results were plotted out as a heatmap with the Pheatmap function for R (https://cran.r-project.org/package=pheatmap). Differential expression analysis of both, known and novel miRNAs was performed with the sRNAde web tool included in the sRNAtoolbox collection [17]. DESeq2 and EdgeR were the methods used by the program. Three different comparisons were performed: Asymptomatic vs Seronegative, Lesions vs Seronegative and Lesions vs Asymptomatic. For a miRNA to be considered differentially expressed (DE), the adjusted *p* value was set to 0.05 and the absolute log_2_ expression fold change (FC) to one.

### Target prediction, gene ontology and pathway analysis

Target genes for each differentially expressed miRNA were predicted using TargetScan 7 [18] and miRanda – via the miRNAconstarget tool included in sRNAtoolbox [17] – algorithms. The 3’ UTR mRNA sequences of sheep for both programs were obtained from the multi-species alignment generated from human 3’ UTRs given by the authors of TargetScan. The threshold for this program was set to absolute context++ score > 1 and the thresholds for miRanda were set to a score higher than 155 and a free energy lower than -20 kcal/mol. The consensus targets predicted by both programs were selected.

Viral-targeting miRNAs in the ovine genome were also inferred by using 11 VMV (Visna Maedi Virus) and 5 Caprine Arthritis Encephalitis Virus (CAEV) complete sequences deposited in GenBank database. The program used was standalone miRanda [19].

In order to obtain biological information from the target genes of differentially expressed miRNAs, an enrichment analysis was performed. We built three sets of genes that interacted in our predictions with any of the DE miRNAs in each comparison. Pathway and gene ontology (GO) analysis were carried out with David (https://david.ncifcrf.gov/) web tool. For pathways, KEGG pathway terms were tested and Benjamini multiple test correction value of 0.05 was applied as a threshold. We used Cytoscape version 3.5.1 [20] to build functional networks merging interactions among miRNAs, target genes and enriched pathways. This way, we were able to visualise genes in the selected pathways that are being targeted by dysregulated miRNAs.

### RT-qPCR validation

To validate changes identified by RNA-seq experiment, the relative expression levels of 7 miRNAs (oar-miR-125b, oar-let-7b, oar-miR-181a, oar-miR-148a, oar-miR-21, oar-miR-30c, oar-miR-379-5p) selected based on significant changes seen in Lesions vs Seronegative comparison in the RNA-seq analysis, were verified by qPCR. The U6 snRNA, oar-miR-30d and oar-miR-191 were tested as internal standard controls and the last two were selected for their expression stability in our samples. Additional file 2 shows the list of the amplified miRNAs and the corresponding primer sequences. The expression study has been based on the analysis of miRNA expression with Fludigm’s BioMark HD Nanofluidic qPCR System technology combined with GE 48.48 Dynamic Arrays IFC. qPCR was performed on a BioMark HD System using Master Mix SsoFastTM EvaGreen® Supermix with Low ROX (Bio-Rad Laboratories, Hercules, CA, USA). The analysis of expression with the Fluidigm Biomark HD Nanofluidic qPCR system was performed at the Gene Expression Unit of the Genomics Facility, in the General Research Services (SGIKER) of the UPV/EHU.

The software for the real-time PCR analysis and obtaining of the Ct values was Fluidigm Real-Time PCR Analysis Software [v3.1.3]. PCR efficiency calculation and correction, reference miRNA stability analysis and normalization was done with GenEx software of MultiD [v5.4]. Most miRNAs showed high amplification efficiencies (94.43–99.65%). The stability of candidate reference miRNAs was analyzed using both NormFinder [21] and GeNorm [22] algorithms integrated in GenEx. The two most stable miRNAs were oar-miR-30d and oar-miR-191 so normalization was performed using these two reference miRNAs. Normal distribution was checked using the Shapiro-Wilk test in the IBM SPSS statistical package [v24]. Comparison and correlation between the RNA-seq and qPCR results was performed using T-test and Pearson’s correlation, respectively. In all analyses, differences were considered significant when *p* values were < 0.05.

## Results

### Small RNA sequencing and miRNA prediction

In the present study, the small RNAs from lung tissue of sheep with and without VMV infection were sequenced. The raw reads were high quality – only approximately 2% had Q scores below 30 – and the numbers of reads ranged from 22 to 8 million, with an average of 15 million reads. The raw reads were analyzed by sRNAbench for miRNA prediction, trimmed the adapters in around the 95% of the reads in all the samples, and 85% of the preprocessed reads were successfully mapped to the sheep genome. The read-length distribution showed a clear peak between 21 and 23 nucleotides in all of the samples, where most of the reads were located.

**Fig. 2:**
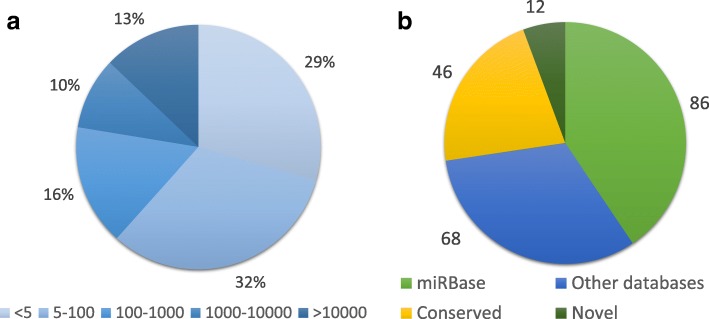
Statistics of RNA-seq and prediction data. **a** Average counts distribution of all the miRNAs detected and predicted. **b** Distribution of miRNAs according to previous knowledge about them

Out of the mapping, the program could annotate 86 known sheep miRNAs from miRBase. All of the other reads that mapped to the genome, but that did not coincide with a miRBase miRNA were subjected to novel discovery tests, from which several new miRNAs arose. Some of these new miRNAs were apparently completely novel molecules, and others were found to be conserved in other species. After cleaning the output sequences and aligning them with RNAcentral, it was found that some were already annotated in sheep and that others had homologs in other species. In total, 86 known miRNAs from miRBase, 68 known sheep miRNAs from other databases, and 58 miRNAs shown for the first time in sheep were found (Fig. 2b). Twelve miRNAs out of these 58 could not be considered ovine homologs of previously described miRNAs and were considered novel. The full list of the described miRNAs not present in miRBase is in Additional file 3. The novel miRNAs were named sequentially, but they were given the name of a homolog if one existed. Regarding the expression levels, some miRNAs were much more abundant than others (Fig. 2a): the 13% most abundant miRNAs were above 10,000 counts, while the 29% least abundant miRNAs had fewer than five average counts. Furthermore, the miRNAs classified as novel or conserved had particularly low abundance, with only few of them having more than 1000 counts.

### Differentially expressed miRNAs

**Fig. 3:**
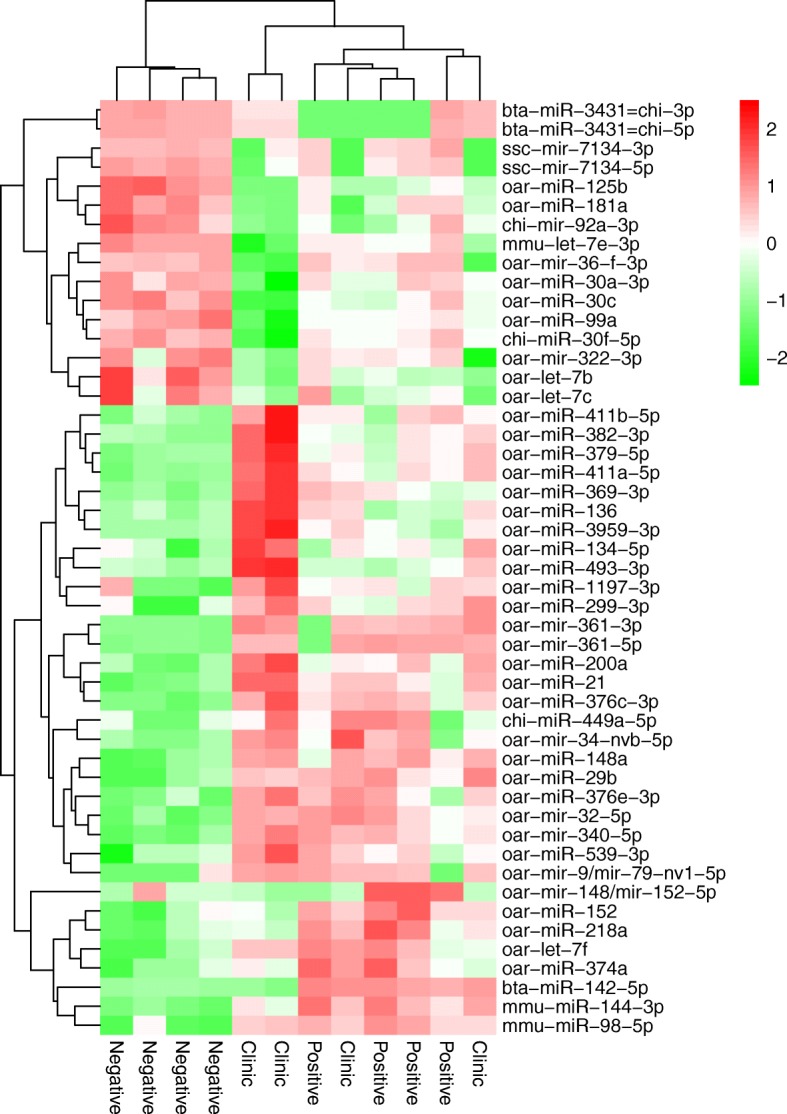
Hierarchical clustering heatmap. Clustering of all the DE miRNAs detected by any of both programs (DESeq2 or EdgeR) and samples. Colours and intensities depend on expression level. Green indicates gene down-regulation and red up-regulation

We made pairwise comparisons among the three sample groups. Overall, the differential expression levels, as well as the PCA, pointed out that the biggest differences were between seronegative sheep and the other two seropositive groups (asymptomatic animals and animals with Lesions). Clustering of differentially expressed (DE) miRNAs detected by either of the two programs clearly grouped the seronegative samples, but failed to distinguish the other two groups, similar to the outcome of the PCA. Seropositive asymptomatic animals and animals with developed clinical symptoms seemed quite similar in terms of miRNA expression (Fig. 3; Additional file 1). By merging the results of the EdgeR and DESeq2 analyses, 34 DE miRNAs were identified between clinically affected and seronegative sheep, of which 23 were upregulated and 11 downregulated. There were also 9 upregulated and one downregulated miRNAs when comparing samples from seropositive asymptomatic animals with samples from seronegative animals, and only three miRNAs were differentially expressed between animals with clinical symptoms and seropositive asymptomatic animals (Table 2). Some novel ovine miRNAs with homologs in other mammals, namely, chi-miR-30f-5p, chi-miR-449a-5p, mmu-let-7e-3p, mmu-miR-144-3p, bta-miR-142-5p, chi-mir-92a-3p, ssc-mir-7134-3p, ssc-mir-7134-5p and mmu-miR-98-5p, from goat (chi), mouse (mmu), pig (ssc) and cattle (bta), showed differences in VMV infected animals. Completely novel miRNAs did not differ significantly in their expression likely due to their low expression levels, which were sometimes even below the applied count threshold.

**Table 2:** Differential expression results of the three comparisons. Only detections by both programs are showed and for the selection, the adjusted *p* values of each program were used. Log_2_ FC of 7.000 in DESeq2 means that the miRNA was present in one group of samples but not in the other

miRNA Name	DESeq2		EdgeR	
	Log_2_FC	Padj	Log_2_FC	FDR
Lesions-Seronegative				
oar-mir-322-3p	−2.830	0.003	−2.880	0.005
chi-miR-30f-5p	−2.199	0.0001	−2.226	0.02
oar-mir-361-3p	7.000	5.64E-13	14.182	3.1E-09
oar-mir-361-5p	7.000	4.57E-17	13.137	3.11E-08
chi-miR-449a-5p	6.270	0.017	6.105	0.009
mmu-let-7e-3p	−2.092	0.029	−2.149	0.008
mmu-miR-144-3p	2.824	0.002	2.792	0.0007
oar-mir-32-5p	3.939	3.45E-05	3.877	0.0001
oar-mir-340-5p	1.827	0.029	1.746	0.038
oar-mir-34-nvb-5p	1.575	0.002	1.500	0.003
bta-miR-142-5p	7.000	3E-10	12.956	3.79E-05
oar-mir-9/mir-79-nv1-5p	4.173	0.0002	4.045	0.043
chi-mir-92a-3p	−1.559	0.0002	− 1.609	0.008
oar-mir-36-f-3p	7.000	2.84E-09	−14.383	3.84E-06
ssc-mir-7134-3p	−3.517	0.0005	−3.676	0.009
ssc-mir-7134-5p	−3.967	0.0002	− 3.968	4.45E-05
oar-let-7b	−1.458	5.28E-05	−1.519	0.002
oar-let-7f	1.156	0.003	1.078	0.003
oar-miR-125b	−2.083	2.65E-08	−2.134	3.84E-06
oar-miR-134-5p	3.267	0.026	3.184	0.002
oar-miR-136	2.623	0.047	2.529	0.003
oar-miR-148a	1.579	2E-05	1.501	1.93E-06
oar-miR-181a	−1.781	1.16E-05	−1.836	6.47E-05
oar-miR-200a	1.614	0.001	1.533	0.008
oar-miR-21	3.584	2E-05	3.500	8.64E-16
oar-miR-299-3p	2.910	0.025	2.726	0.008
oar-miR-29b	2.156	0.01	2.087	0.016
oar-miR-30c	−1.440	0.0002	−1.486	0.003
oar-miR-369-3p	2.966	0.043	2.858	0.002
oar-miR-376c-3p	2.268	0.022	2.158	0.002
oar-miR-376e-3p	2.058	0.032	1.975	0.006
oar-miR-379-5p	2.336	0.017	2.237	0.008
oar-miR-411a-5p	2.181	0.008	2.084	0.026
oar-miR-99a	−1.117	0.002	−1.168	0.025
Seropositive asymptomatic-Seronegative				
oar-mir-361-3p	7.000	7.11E-10	11.903	5.25E-07
oar-mir-361-5p	7.000	2.08E-17	13.286	6.77E-08
mmu-miR-144-3p	3.733	6.95E-05	3.690	8.51E-06
oar-mir-32-5p	3.679	0.0002	3.634	0.0005
bta-miR-142-5p	7.000	2.42E-11	13.831	1.67E-05
mmu-miR-98-5p	5.023	0.0002	5.006	0.029
oar-let-7f	1.303	0.0007	1.237	0.0005
oar-miR-125b	−1.361	0.0007	−1.401	0.008
oar-miR-148a	1.297	0.0009	1.224	0.0002
oar-miR-21	2.099	0.026	2.029	5.99E-06
Lesions-Seropositive asymptomatic				
oar-mir-148/mir-152-5p	−4.143	0.032	−4.098	0.005
oar-mir-36-f-3p	7.000	6.99E-08	−13.615	6.42E-05
ssc-mir-7134-5p	−3.016	0.044	−2.973	0.009

**Fig. 4:**
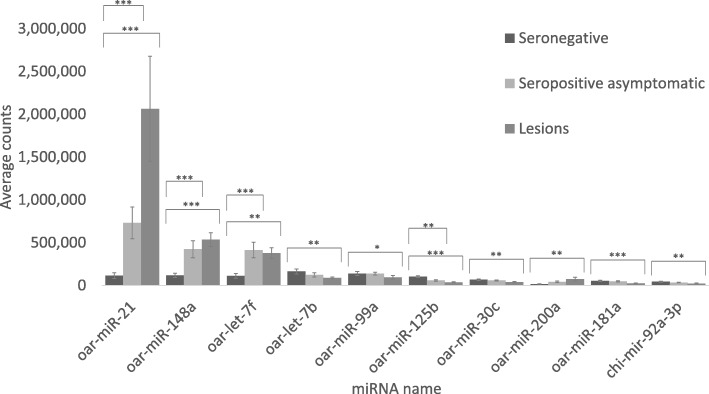
Expression of most abundant miRNAs. Average counts of the most expressed DE miRNAs in the three phases of disease progression. Asterisks indicate significance level between two groups (*P < 0.05, **P < 0.01, ***P < 0.001)

Among the most abundantly expressed DE miRNAs, some showed relevant increases or reductions in expression (Fig. 4): oar-miR-21 was, by far, the most abundant DE miRNA, since its expression was elevated 4.3 times in seropositive asymptomatic animals and 12 times in diseased animals, with average total counts of around two million. Other highly expressed DE miRNAs, such as oar-miR-148a and oar-let-7f showed significant increases, with absolute fold changes of 3 and 2.2, respectively, in infected animals compared with seronegative animals. Furthermore, miRNAs such as oar-let-7b, oar-miR-99a and oar-miR-125b, showed reduced expression in infected sheep (Fig. 4).

### Validation of differential miRNA expression

**Fig. 5:**
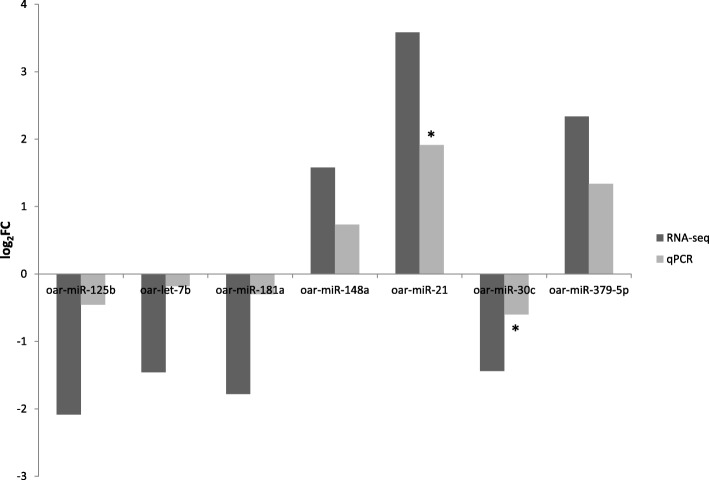
qPCR validation of miRNAs. Expression of selected miRNAs in Lesions group relative to Seronegative group measured by RNA-seq and qPCR. Bars represent the average results of the different samples. Statistically significant differences in the expression measured by qPCR of the indicated miRNAs are showed with an asterisk (*p* < 0.05)

To validate the miRNA-seq data, seven miRNAs (oar-miR-125b, oar-let-7b, oar-miR-181a, oar-miR-148a, oar-miR-21, oar-miR-30c, and oar-miR-379-5p) were verified using the Fluidigm Biomark HD Nanofluidic qPCR system. The log_2_FC in the miRNA expression levels calculated by qPCR in the Lesions group relative to the Seronegative group are shown in Fig. 5. The validation results confirmed the upregulated expression of 3 miRNAs (oar-miR-148a, oar-miR-21, oar-miR-379-5p) and the downregulated expression of 4 miRNAs (oar-miR-125b, oar-let-7b, oar-miR-181a, and oar-miR-30c), although only two were statistically significant: oar-miR-21 (*p* = 0.003) and oar-miR-30c (*p* = 0.004). There were no significant differences in the FC data obtained from the RNA-seq and the Fluidigm Biomark HD Nanofluidic qPCR system (*p* = 0.656) showing a high degree of concordance, with a correlation coefficient of 0.982 (*p* = 0.000).

### Functional analysis of dysregulated miRNAs

In this study, the targets of the DE miRNAs were predicted using the TargetScan and Miranda algorithms. TargetScan predicted a total of 1.9 million interactions for all of the identified miRNAs, and this number was reduced to 124,614 after applying the cut-off value. Miranda predicted 911,069 target sites for the same set of miRNAs and application of the threshold settings reduced this number to 41,871 targets. Next, we performed an intersection analysis to enhance the confidence of the predictions, and this process reduced the number of interactions to 12,280, with 6426 unique genes. An average of 35 interactions was observed for each of the 349 mature miRNAs analyzed. Out of the collection of the predicted targets, we retrieved three sets of genes (one for each comparison) with 1736, 1135 and 190 genes each. These gene sets were then used in enrichment analyses.

The GO enrichment analysis did not identify any significantly enriched terms using the multiple testing correction, whereas some pathways were actually overrepresented, such as, signalling pathways (e.g. PI3K-Akt, AMPK and ErbB), or other terms such as ECM-receptor interaction and pathways in cancer (Table 3). The PI3K-Akt signalling pathway had the most genes involved in both comparisons – 51 and 40, respectively – and it was the most statistically significant term (corrected *P* values of 2.51E-04 and 0.004). The comparisons between the seropositive and seronegative sheep were the only ones yielding results, while there were no enriched terms in the comparison between the seropositive groups, based on the corrected *p* values.

**Table 3:** Enrichment analysis of pathways between both seropositive groups and the seronegative group. Significant entries with Benjamini score equal or smaller than 0.05 are shown

Pathway	Seropositive asymptomatic-Seronegative		Lesions-Seronegative	
	Fold enrichment	Benjamini	Fold enrichment	Benjamini
oas04151:PI3K-Akt signaling pathway	2.327	2,51E-04	1.868	0.004
oas04152:AMPK signaling pathway	2.831	0.024	2.411	0.022
oas05202:Transcriptional misregulation in cancer	–	–	2.111	0.024
oas05161:Hepatitis B	2.542	0.027	2.134	0.047
oas04012:ErbB signaling pathway	3.296	0.034	2.519	0.048
oas05200:Pathways in cancer	–	–	1.595	0.049
oas04512:ECM-receptor interaction	3.186	0.024	2.435	0.050
oas04510:Focal adhesion	2.232	0.029	–	–
oas05215:Prostate cancer	3.296	0.034	–	–
oas04360:Axon guidance	2.643	0.034	–	–
oas04014:Ras signaling pathway	2.112	0.038	–	–
oas05206:MicroRNAs in cancer	2.190	0.050	–	–

**Fig. 6:**
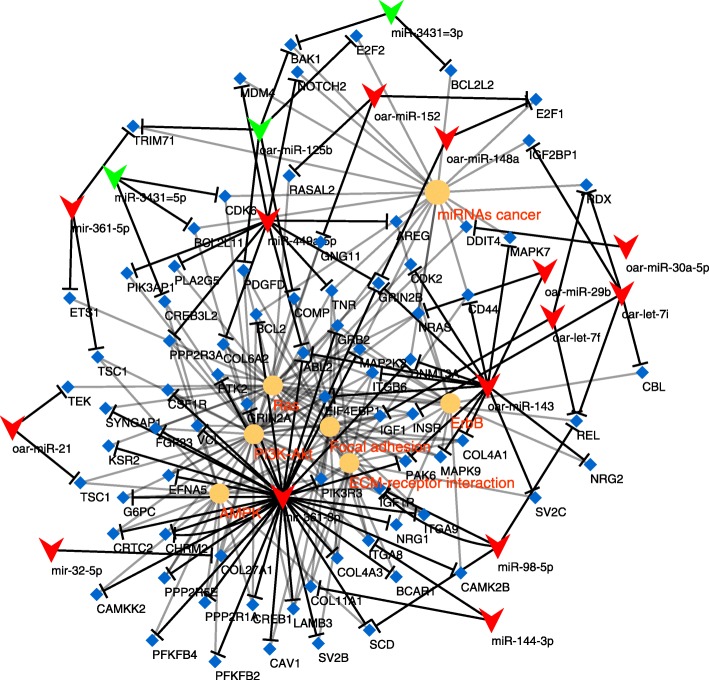
Functional network of the comparison between seropositive asymptomatic and seronegative sheep. It illustrates the predicted interactions of DE miRNAs with their targets and the pathways those target genes are part of. Upregulated miRNAs are coloured in red and downregulated ones in green, pathway names in orange and genes in blue

**Fig. 7:**
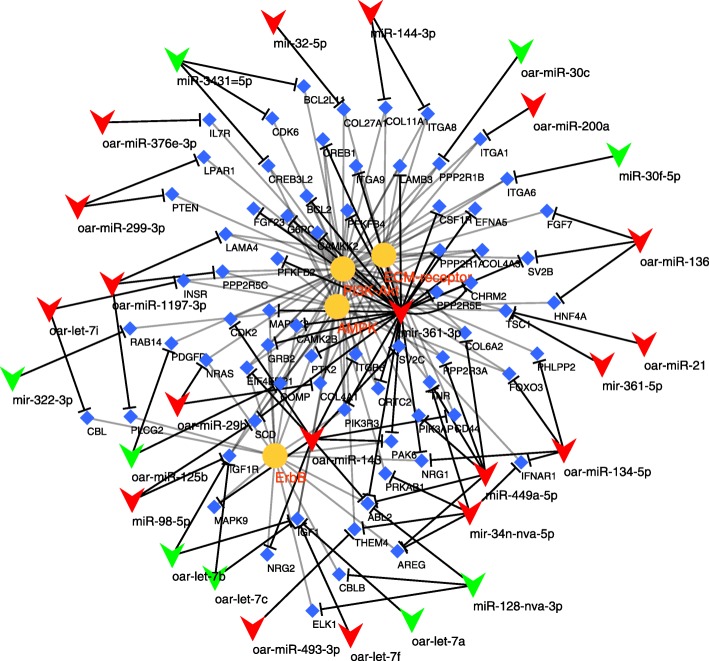
Functional network of the comparison between diseased and seronegative sheep. It illustrates the predicted interactions of DE miRNAs with their targets and the pathways those target genes are part of. Upregulated miRNAs are coloured in red and downregulated ones in green, pathway names in orange and genes in blue

Interaction maps incorporating the miRNAs and their targets and the pathways information were produced in an attempt to unveil how the differences in miRNA expression could affect these pathways in seropositive asymptomatic compared to seronegative animals (Fig. 6) and in diseased animals compared to seronegative animals (Fig. 7). Key regulators in the PI3K-Akt pathway, such as PTEN, and related transcription factors such as FOXO3 and CREB1, appear to be targeted by dysregulated miRNAs identified between the seropositive groups and the seronegative group. Most of the miRNAs target no more than three genes in these pathways, except for oar-miR-143 and oar-mir-361-3p, which target several genes based on our predictions.

### Virus-miRNA interactions

Regarding the highly expressed DE miRNAs, two significantly strong interactions were found between the miRNAs and the SRLV genome. The upregulated miRNA oar-miR-200a was predicted to target nine out the eleven tested sequences at nucleotides 1671 to 1689 with respect to the VMV reference genome sequence (GenBank accession number L06906.1), with a score of 155 and a folding energy of − 16.1 kcal/mol. The downregulated miRNA oar-miR-99a was predicted to target nine sequences around nucleotides 5383 to 5402 with a score of 150 and a folding energy of − 25.54 kcal/mol. These predicted interactions are in the “gag” and “vif” genes, respectively. These targeted sequences are all from the genotype A of SRLV. On the other hand, oar-miR-99a may also target CAEV at nucleotides 2194 to 2212 – in the “pol” gene – with respect to the CAEV reference genome (GenBank accession number M33677.1) with a score of 160 and a folding energy of − 23.83 kcal/mol.

## Discussion

In this work, we used NGS techniques to analyze the expression pattern of miRNAs in seronegative sheep and in SRLV seropositive but asymptomatic animals and in diseased animals. We then made predictions of the possible regulatory functions of the miRNAs. Since we used tissue samples from naturally infected animals for the experiments, the data reflect the actual miRNA transcriptome in the lung tissue of SRLV-infected animals. Host-virus interactions modify several biological processes as a consequence of the ability of the viruses to employ the host machinery to complete their replication cycle, and of the host’s attempts to deal with the infection. These changes can be observed at the miRNA expression level since miRNAs can control different pathways; therefore, understanding changes in miRNA expression could be crucial for understanding the disease.

The enriched pathways identified in this study suggest an increase in cell proliferation-related signaling. The PI3K-Akt pathway is a key pathway involved in growth and proliferation, and it has been extensively studied in the context of proliferative diseases such as cancer; furthermore, it seems to be influenced by a miRNA regulatory network as an added layer of modulation [23]. Furthermore, viruses can hijack this pathway for enhanced replication, as has been reported in several cases [24]. For instance, Porcine Reproductive and Respiratory Syndrome Virus (PRRSV) modulates PI3K-Akt signalling via FoxO1 and Bad ([25]) and influenza A codes for the NS1 protein which directly interacts with the PI3K regulatory subunit p85 ([26]). DE miRNAs were predicted to target very important factors in this pathway including PTEN, PI3K, FOXO3, the BCL2 family, CREB, GRB2, growth factors (FGF23) and cytokine receptors (IFNAR1). Other enriched pathways in our set of target genes were the AMPK signalling pathway, which is a regulator of cellular homeostasis and is linked to PI3K-Akt pathway, and the ErbB pathway, which is related to signal transduction involving growth factors.

Although miRNAs are fine tuners of gene expression that can act at low concentrations, the appearance of highly expressed miRNAs may be very relevant and could indicate strong modulation. Normally, a few miRNAs comprise the majority of the miRNAome, and many others are present at low concentrations. In our experiments, oar-miR-21 expression showed an interesting behaviour, as its expression is remarkably high in both seropositive groups, with its highest expression level in diseased animals. miR-21 is a fairly well-studied miRNA, and was one of the first miRNAs identified as an oncogene; it has been seen to be upregulated in several conditions including tumours [27] and viral infections. In the case of RNA viral diseases, miR-21 is upregulated by hepatitis C virus (HCV), which leads to a decreased IFN response in human cell lines [28], during dengue virus infection in human cancer cells, which promotes viral replication [29] and in HIV and in HIV-related pulmonary arterial hypertension in human plasma [30]. Furthermore, Epstein-Barr virus (EBV) induces miR-21 expression in B cells, which promotes tumorigenesis by activating the PI3K-Akt pathway, causing FOXO3a to stop repressing miR-21 [31, 32], findings that are in agreement with our current results.

The respiratory form of SRLV infection exhibits some typical histopathological lesions characterized by lymphocytic infiltration and inflammation, M2-polarized macrophages, interstitial pneumonia, lung fibrosis and decreased gas exchange [33, 34]. However, the mechanisms of this pathogenesis, which are likely immunomediated [35], are not fully characterized. There were no major differences between the infected asymptomatic animals and the sheep that did show lesions, indicating that the miRNA levels mostly change after infection, rather than when symptoms appear. It seems that most of the transcriptional changes occur in the early stages of infection and that the differences between the asymptomatic-seronegative and the lesions-seronegative comparisons could be due to disease progression and appearance of clinical symptoms.

Interestingly, these kinds of lesions could be related to some of the DE miRNAs and with the pathways regulated by them. In an artificially induced lung fibrosis in mice, miR-21 mediates the activation of pulmonary fibroblasts [36]. Furthermore, miR-21 has been recently proposed as an indicator of disease progression and potential treatment target in another mouse model [37]. MiR-21 could control pathways such as the TGF-β1 signaling pathway by targeting SMAD7 and SPRY1 or by inhibiting PTEN, which is a known negative regulator of lung fibrosis [38]. The remodelling of lung tissues caused by fibrosis related hypoxia has also been linked with miR-21 [39]. Importantly, PTEN has a crucial role in controlling the PI3K-Akt pathway, and its interaction with miR-21 has been experimentally validated several times in human and in mice [40]. The upregulated miR-148a also targets PTEN, as well as GADD45A and BCL2L11, and it accelerates the development of autoimmunity [41].

Another miRNA, miR-99a, which was downregulated in the diseased sheep, appears to target AKT1 [42] (which has an important role in the PI3K-Akt pathway) and inhibits cancer cell proliferation by targeting mTOR [43]. Thus, its downregulation in the animals with lesions should increase AKT1 and mTOR expression, stimulating proliferative signal. In our analysis, inflammation-related interleukin 13 (IL-13) was predicted as a target of miR-98-5p and let-7 family miRNAs, and it is noteworthy that previous experimental observations have shown that let-7 miRNAs can modulate inflammation through inhibition of IL-13 [44]. During bluetongue virus infection in sheep testicular cells, while IL-13 and let-7f were downregulated, let-7d was upregulated and PI3K-Akt pathway was overrepresented in the enrichment test of the DE genes [45].

The relationship between the dysregulation of some miRNAs and VM disease could be a direct consequence of virus modulation or a side effect of the host defense mechanisms. In the case of miR-21, it has been proposed as a key switch in the inflammatory response [40]. Clinical lesions observed could be a consequence of excessive cell survival signalling after the initial proinflammatory immune response. On the other hand, the virus itself may modulate miRNA expression, as it does in EBV and HCV infections [28, 46], during which the viruses induce miR-21 expression to promote their replication by enhancing the growth and survival of the infected cells, thus modulating the response in favour of the virus. Furthermore, PRRSV downregulated miR-125b to negatively regulate NF-κB signaling as a survival strategy [47].

Direct targeting of viruses remains controversial not only because of viral genome structure and rapid evolution but also because the normal concentrations of miRNAs are too low for efficient silencing [48]. Only some highly expressed DE miRNAs have been analyzed to determine if they could potentially silence some viral RNA. Interestingly, there were some predicted miRNA target sites in the SRLV genome, including one for oar-miR-200a. oar-miR-200a was upregulated in the lesions-seronegative comparison and could actively target the viral *gag* gene in the A genotype. Functional experiments are necessary to uncover the antiviral functions of these candidate miRNAs.

## Conclusions

In this work, we performed for the first time a miRNA profiling in sheep responding to SRLV infection. Twelve completely novel miRNA molecules and more than 40 others were found for the first time in sheep. MiRNAs differentially regulated between seronegative and infected sheep, such as oar-miR-21, oar-miR-148a or oar-let-7f may have potential implications for the host-virus interaction. The miRNAs were predicted to target important genes involved in apoptosis, proliferation and growth, e.g., the PI3K-Akt and AMPK pathways. The role of oar-miR-21 as a regulator of inflammation and proliferation appeared as a possible cause for the lesions caused in sheep lungs, and this miRNA could be an indicator of the severity of the lung lesions or may be useful as a putative target for therapeutic intervention.

## Additional files

Additional file 1: PCA analysis of the 12 samples used in the RNA-seq analysis. (PDF 25 kb)

Additional file 2: Primers used for RT-qPCR validation of selected miRNAs (XLSX 8 kb)

Additional file 3: List of described miRNAs not present in miRBase (XLSX 16 kb)

## Abbreviations

CAEV: Caprine arthritis encephalitis
DE: Differentially expressed
EBV: Epstein-barr virus
ELISA: Enzyme-linked immunoSorbent assay
FC: Fold change
GO: Gene ontology
HCV: Hepatitis C virus
miRNAs: MicroRNAs
PCA: Principal component analysis
PRRSV: Porcine reproductive and respiratory syndrome virus
RISC: RNA-induced silencing complex
SRLVs: Small Ruminant Lentiviruses
TLRs: Toll like receptors
VM: Visna/Maedi disease
VMV: Visna maedi virus
