# Supplementary figures and images for: Expression analysis of lung miRNAs responding to ovine VM virus infection by RNA-seq
Source: BMC Genomics. 2019 Jan 18;20:62. doi: 10.1186/s12864-018-5416-0 (PMC6339376; doi:10.1186/s12864-018-5416-0)

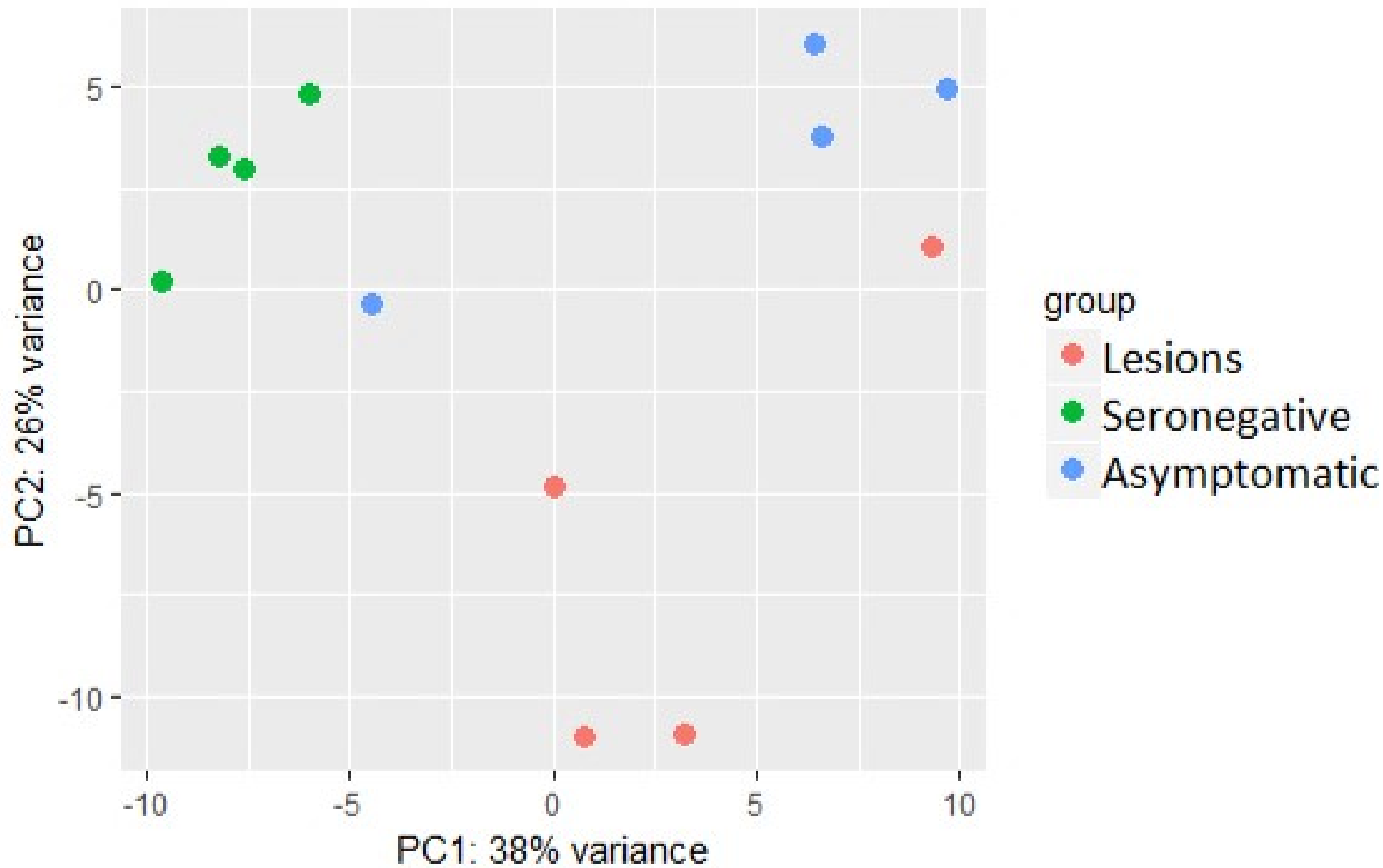

Supplement: Supplementary file 1 — PCA analysis of the 12 samples used in the RNA-seq analysis. (PDF 25 kb) [file 12864_2018_5416_MOESM1_ESM.pdf]
